# Editorial: Computational Neuroscience for Perceptual Quality Assessment

**DOI:** 10.3389/fnins.2022.876969

**Published:** 2022-03-28

**Authors:** Xiongkuo Min, Ke Gu, Lu Zhang, Vinit Jakhetiya, Guangtao Zhai

**Affiliations:** ^1^Department of Electronic Engineering, Shanghai Jiao Tong University, Shanghai, China; ^2^Faculty of Information Technology, Beijing University of Technology, Beijing, China; ^3^Univ Rennes, INSA Rennes, CNRS, IETR - UMR 6164, Rennes, France; ^4^Department of Computer Science Engineering, Indian Institute of Technology, Jammu, India

**Keywords:** computational neuroscience, perceptual quality assessment, perception, multimedia, image processing

Quality assessment aims to measure the degree of delight or annoyance of the users when experiencing an application or service. With the quick improvement of content acquisition, processing, transmission, and display techniques, the end-users are expecting and demanding continuously improved quality of experience (QoE) from the service providers. To guarantee a good QoE to end-users, perceptual quality assessment is introduced and widely studied in recent years (Brunnström et al., 2013; Zhai and Min, 2020; Min et al., 2022). Since the ultimate receiver of the processed signal is usually human, it is reasonable and beneficial to take human perception properties into consideration. Though we still have limited knowledge of the intrinsic neuroscience working mechanism of human perception, it is worthwhile to study and take inspiration from neuroscience and utilize these properties for computational modeling of perceptual quality.

Many of the current quality assessment models have already attempted to include human perception properties at some level, however, the majority of these models only take simplified concepts of human perception, and use “black box” machine learning techniques to model the QoE. The rapid development of neuroscience and computer science have provided opportunities for deeper explorations of the intrinsic neuroscience working mechanism of quality perception, and to utilize computational neuroscience theories and models for more efficient and explainable quality assessment. Specifically, on one hand the underlying biological bases of human perception especially those related to quality perception can be further explored on the basis of the recent advancement of neurobiology. While on the other hand, it is worthwhile to seek better ways to apply the relevant neuroscience working mechanisms for quality assessment and to build more accurate brain-inspired computational quality assessment models.

This Research Topic is a collection of articles concerning computational neuroscience studies for perceptual quality assessment and the potential applications in artificial systems. The final list of accepted articles can be categorized into four groups: 1. Neuroscience studies of human perception, especially those related to quality perception; 2. Neuroscience inspired perceptual quality modeling; 3. Perceptual quality assessment for emerging and advanced multimedia technologies; 4. Applications of perceptual quality modeling. The below is an overview and discussion of the accepted articles.

## Neuroscience Studies of Human Perception, Especially Those Related to Quality Perception

In recent years, a large amount of perceptual quality assessment studies has taken human perception properties into consideration, since human is usually the ultimate judger of signal quality. To study the intrinsic neuroscience working mechanism of human perception, subjective neuroscience and perceptual studies are usually necessary.

The influence of audio on perceptual QoE has been studied and verified by some previous studies (You et al., 2010; Akhtar and Falk, 2017; Min et al., 2017, 2020a,b). In this Research Topic, Sun and Hines give an overview for the audiology and cognitive science researches which study how cognitive processes influence the quality of listening experience. Moreover, they also propose to introduce these mechanisms from audiology and cognitive science into the current QoE framework, through which we can better incorporate cognitive load in speech listening. Pieper et al. use electroencephalogram and some other questionnaire-based subjective measures to study if noise-canceling technologies can reduce the influence of external distractions and free up mental resources. Results partially verify that an assumed lower mental load is observed in no noise and noise-canceling environment compared to that of in the noise environment. Han et al. study the influence of the refresh rate of a display on the motion perception response. Moreover, they introduce an objective visual electrophysiological assessment model to better select the display parameters.

## Neuroscience Inspired Perceptual Quality Modeling

Full understanding of the intrinsic neuroscience working mechanism of human perception is difficult in the current stage, however it is worthwhile to study and take inspiration from neuroscience and utilize these properties for computational modeling of perceptual quality.

Over the last two decades, many perceptual quality assessment models have been proposed (Wang et al., 2004; Brunnström et al., 2013; Min et al., 2018a,b, 2022; Zhai and Min, 2020), and many of them have taken inspirations from neuroscience. Song et al. introduce a blind quality assessment model for authentically distorted images by considering both distortion degree and intelligibility. Specifically, they analyze the relation between intelligibility and image quality, and then incorporate such intelligibility into a highly generalizable image quality prediction model. Feng et al. introduce an end-to-end cross-domain feature similarity guided deep neural network for perceptual quality assessment. This model is built based on the observation that features for the object recognition task and features for the quality prediction task are highly correlated in terms of characteristics of the human visual system. Experimental results have verified the effectiveness of the proposed model.

## Perceptual Quality Assessment for Emerging and Advanced Multimedia Technologies

Recently, a growing number of emerging and advanced multimedia technologies or systems have invaded into our daily lives, for example light field, virtual reality, etc. Such emerging multimedia applications also call for new quality perception models, since traditional quality perception models are not good at such contents.

In this Research Topic, Meng et al. propose a light field image quality assessment model by predicting the global angular-spatial distortion of macro-pixels as well as the local angular-spatial quality of the focus stack. Wang et al. present a quality metric for depth-image-based rendering images by jointly measuring the synthesized image's colorfulness, texture structure, and depth structure. Hu et al. first introduce a method to simulate the wrap-around artifact on the artifact-free MRI image to increase the quantity of MRI data, and then propose an image restoration method to reduce the wrap-around artifact.

## Applications of Perceptual Quality Modeling

The research of perceptual quality modeling applications has also aroused increasing attention in recent years, since perceptual quality modeling can play an important role in the quality control and optimization of multimedia communication systems. In this Research Topic, Lei et al. first introduce a new quality assessment database for swimming pool images, and then propose an objective swimming pool image quality measure by detecting the main target and integrating multiple quality-aware features. Yu et al. first construct a new image database by collecting 1,000 pictures from the official social network accounts of nine well-known universities, as well as the corresponding number of page views.

We hope that readers find this Research Topic useful, timely and informative, in addressing the important topics in Computational Neuroscience for Perceptual Quality Assessment.

## Author Contributions

All authors wrote, and equally contributed to the article, and approved the submitted version.

## Conflict of Interest

The authors declare that the research was conducted in the absence of any commercial or financial relationships that could be construed as a potential conflict of interest.

## Publisher's Note

All claims expressed in this article are solely those of the authors and do not necessarily represent those of their affiliated organizations, or those of the publisher, the editors and the reviewers. Any product that may be evaluated in this article, or claim that may be made by its manufacturer, is not guaranteed or endorsed by the publisher.
